# A Relatively Small Gradient of Extracellular pH Directs Migration of MDA-MB-231 Cells In Vitro

**DOI:** 10.3390/ijms21072565

**Published:** 2020-04-07

**Authors:** Eiji Takahashi, Daisuke Yamaguchi, Yoshihisa Yamaoka

**Affiliations:** Graduate School of Advanced Health Sciences, Saga University, Honjyo Machi 1, Saga 840-8502, Japan

**Keywords:** metastasis, cell migration, pH gradient, oxygen gradient, MDA-MB-231 cells

## Abstract

Hematogenous tumor metastasis begins with the invasion and spread of primary tumor cells in the local tissue leading to intravasation. We hypothesized that tumor cells might actively migrate toward intratumor vessels with the extracellular metabolic gradient acting as a guiding cue. Here, we determined in vitro whether the extracellular gradient of pH can act as a cue for directional migration in MDA-MB-231 cells. Cell migration was determined by the wound-healing assay under gradients of extracellular pH (~0.2 units/mm) and oxygen concentration (~6% O_2_/mm) that were produced by a microfluidic device, gap cover glass (GCG). Without GCG, the migration of cells was spatially homogeneous; the same number of cells migrated to the rectangular wound space from the left and right boundaries. In contrast, when GCG generated pH/O_2_ gradients across the wound space, the number of cells migrating to the wound space from the boundary with higher pH/O_2_ values was considerably decreased, indicating a preferential movement of cells toward the region of higher pH/O_2_ in the gradient. The addition of hepes in the extracellular medium abolished both the extracellular pH gradient and the directional cell migration under GCG. We conclude that relatively small gradients of pH in the extracellular medium compared to those found in Na^+^/H^+^ exchanger-driven cell migration were sufficient to guide MDA-MB-231 cells. The directional cell migration as guided by the metabolic gradient could effectively elevate the probability of intravasation and, ultimately, hematogenous metastasis.

## 1. Introduction

The presence of metastasis is an important prognostic factor in the majority of tumor patients. Distant metastasis begins with the intravasation of primary tumor cells into intratumoral blood and lymphatic vessels. Local tumor cell proliferations elevate hydrostatic pressure in the interstitium, which tends to push tumor cells into microvessels. Tumor angiogenesis and lymphangiogenesis further enhance passive intravasation. In addition to passive cell movement, active cell migrations guided by chemokines are also occurring, in which cancer-associated fibroblasts play a role [1,2,3].

Considering the analogy of chemotaxis, where component movement depends on the concentration gradient of specific chemoattractants, it has long been postulated that the gradients of nutrients and metabolic waste might guide tumor cell migration. This prediction is reinforced given the metabolic characteristics peculiar to solid tumors. In solid tumors, the structures of blood vessels are immature and their topological configuration in tissues is generally irregular [4]. As a result, the convectional and diffusional transports of substances between individual cells and blood are considerably hindered and steep concentration gradients of nutrients and metabolic wastes are established within the tissue [4,5]. Although <0.1% of primary tumor cells succeed in intravasation in the local tissue [6], directional cell migration guided by these metabolic gradients, if any, could elevate the probability of intravasation and, ultimately, hematogenous metastasis. However, evidence indicating the status of extracellular nutrient/metabolite gradients as guiding cues for cancer cell migration is scarce at the present time.

Kedrin et al. [7] followed in vivo migrations of the Dendra2-labeled metastatic breast cancer cell line, MTLn3, in an orthotopic mammary carcinoma mouse model for extended periods of time. After photo-switching Dendra2 fluorescence from green to red in situ, the labeled cells spread randomly in various directions in the local tissue. In contrast, in the presence of a micro blood vessel, the photo-switched cells appeared to preferentially migrate toward the nearby blood vessel. Furthermore, the number of photo-switched cells in this tissue volume was significantly reduced within 24 h. These authors also located photo-switched tumor cells in remote organs such as the lungs. These results are consistent with the hypothesis that an active mechanism attracts tumor cells to nearby blood vessels, eventually leading to intravasation and subsequent distant metastasis. The question still to be answered is what the cue for this directional migration was.

Given the above, our initial hypothesis is that the metabolic gradients in the tissue might be a cue for guiding the tumor cells to nearby blood vessels. Because acidosis and hypoxia are the hallmark of an invasive hypoxic tumor [8,9], we focused, among numerous cues in tissues [10], on H^+^ and oxygen particularly their concentration gradients. Hereafter, we refer to the gradient of pH and oxygen concentration as metabolic gradients.

To test this hypothesis in vitro, we used a glassware product that we previously devised and call gap cover glass (GCG; described below) [11] to apply in producing metabolic gradients in cultured monolayer cells. In fact, we demonstrated that gradients of extracellular pH with a magnitude of 0.2–0.3 units/mm are established at three hours after placing the GCG onto confluent monolayer MDA-MB-231 cells [12], while gradients of oxygen concentration under the GCG has not yet been demonstrated. Furthermore, we demonstrated a tendency that MDA-MB-231 cells migrate toward the opening of GCG; in other words, toward areas of higher pH and/or oxygen concentration. One of the serious drawbacks of our approach, however, was that migrating cells under the GCG frequently underwent collisions with each other and subsequently changed their direction of migration. Thus, we thought that our tentative conclusions regarding directional migration of MDA-MB-231 cells were far from promising. To overcome the collision artifacts, we refined our technique by combining our GCG with the conventional wound-healing assay for cell migration [13]. At this point, we became ready to explore whether MDA-MB-231 cells demonstrate directional migration according to the metabolic gradients produced by GCG. Thus, the specific purpose of the present study was 1. to determine, with new improved technique for assessing cell migratory behaviors, whether MDA-MB-231 cells demonstrate directional migration under the GCG, 2. to clarify the role of pH in migratory behavior of the cell by comparing the migration of MDA-MB-231 cells with and without gradients of extracellular pH, and 3. to determine oxygen concentration gradients under the GCG to address the effect of oxygen gradients in the directional cell migration.

We demonstrated with a new improved technique that MDA-MB-231 cells under GCG migrate toward higher pH/O_2_ regions while directional migration was abolished after eliminating gradients of pH. Thus, relatively small gradients of pH in the extracellular medium compared to those found in Na^+^/H^+^ exchanger-driven cell migration were sufficient to guide MDA-MB-231 cells in vitro.

## 2. Results

### 2.1. Extracellular Gradients of Oxygen Concentration under GCG

**Figure 1:**
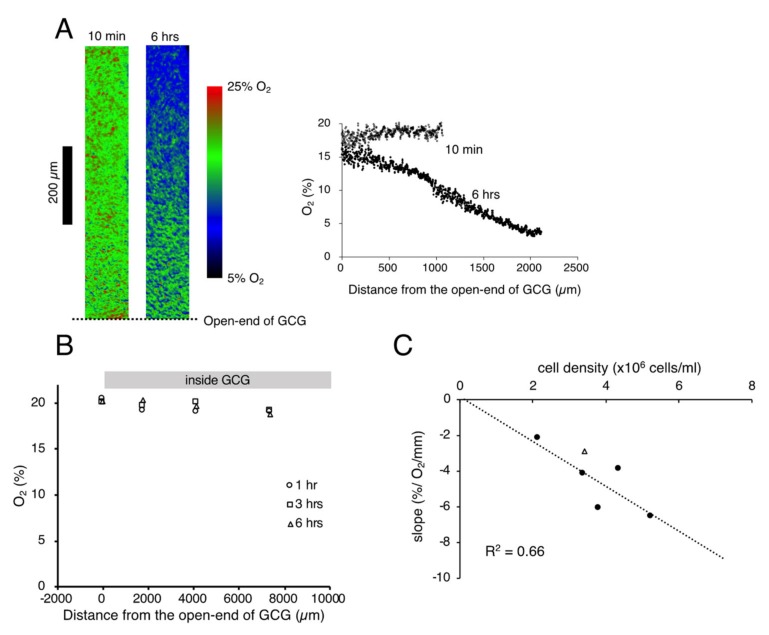
(**A**). Representative data indicating changes in oxygen concentration in the extracellular medium along the oxygen diffusion path as measured by the oxygen sensor foil. The ratio of red and green fluorescence is represented in pseudo-color. At 10 min after placing the gap cover glass (GCG), no gradient was demonstrated underneath. At six hours after placing the GCG, a −6.2% O_2_/mm linear oxygen concentration gradient was produced under the GCG. The number of MDA-MB-231 cells in the culture dish was 5.2 × 10^6^ cells/mL. Note that the oxygen concentration at the opening of the GCG (distance = 0) was substantially lower than that in the microincubator (21%), probably due to the existence of an oxygen diffusion barrier in the bulk medium as suggested by Metzen et al. [14]. (**B**) Effect of mitochondrial respiratory complex III inhibitor (antimycin A, 2 µM) on the oxygen concentration gradient. Elimination of mitochondrial respiration abolished the oxygen concentration gradient under GCG. Although the cell shape changed slightly six hours after respiratory inhibition, the cells were viable as judged by the LIVE/DEAD cell imaging kit (R37601, Thermo Fisher Scientific). The cell density was 5.5 × 10^6^ cells/mL. (**C**) Relationship between the magnitude of the oxygen concentration gradient and cell density. The linear relationship indicates that the oxygen concentration gradient depends upon oxygen consumption of the cell per unit volume. The open triangle represents the slope in which L-15 medium was buffered with 15 mM hepes.

We successfully visualized the oxygen concentration gradient under GCG. As shown in Figure 1A, immediately after placing the GCG, the oxygen concentration along the oxygen diffusion path was constant up to 1200 µm inside the GCG. Subsequently, the oxygen concentration gradients developed slowly and, at six hours after placement of the GCG, a −6.5% O_2_/mm linear gradient was established under GCG. The oxygen concentration gradient abolished after treatment with a mitochondrial respiratory complex III inhibitor (antimycin A, 2 µM), indicating that the oxygen concentration gradient depends upon mitochondrial respiration (Figure 1B). Magnitude of the oxygen concentration gradient increased in proportion to the number of cells per unit volume (Figure 1C). In our culture conditions, 100% confluence corresponded to the cell density of ~5.5 × 10^6^ cells/mL and, therefore, the largest magnitude of the oxygen concentration gradient attainable in our GCG system was ~6% O_2_/mm. It is notable that the oxygen concentration at the entrance of the GCG was substantially lower than the air level. This indicates the presence of an oxygen diffusion barrier in the extracellular medium as suggested previously by Metzen et al. [14].

### 2.2. Effects of Hepes-buffered L-15 on pH and Oxygen Gradients under GCG

**Figure 2:**
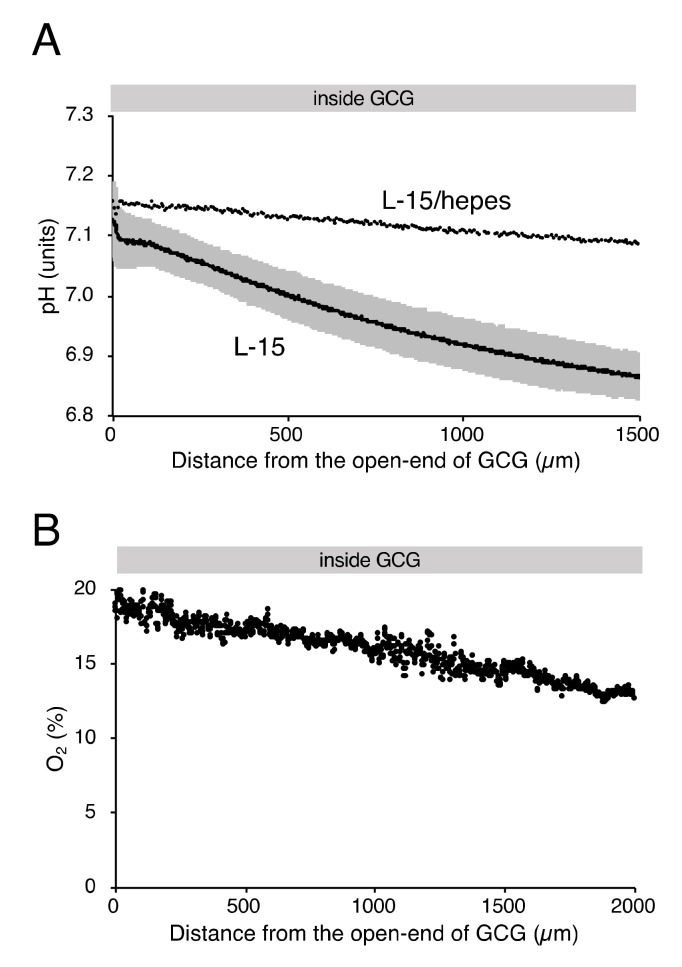
Gradients of extracellular pH and oxygen concentration under GCG in hepes (15 mM)-buffered L-15 medium. (**A**) Representative pH data in L-15/hepes medium. Average pH gradients in L-15 medium are also shown, where the range of ±standard deviation (SD) is represented in gray (n = 4). Data were collected three hours after placing the GCG. (**B**) Representative oxygen gradients in L-15/hepes medium. Cell density-corrected oxygen gradients represented as the open triangle in Figure 1C were comparable to those in L-15 medium. Data were collected six hours after placing the GCG.

As reported elsewhere [12], extracellular pH gradients along the diffusion path slowly developed in GCG and reached the steady state in three hours. Gradients of pH with a magnitude of 0.2–0.3 units/mm were consistently demonstrated (Figure 2A). Supplementing hepes into the L-15 medium abolished the pH gradient (Figure 2A). On the other hand, the addition of hepes in L-15 medium did not affect oxygen concentration gradients under GCG (Figure 2B and the open triangle in Figure 1C).

### 2.3. Cell Migration

**Figure 3:**
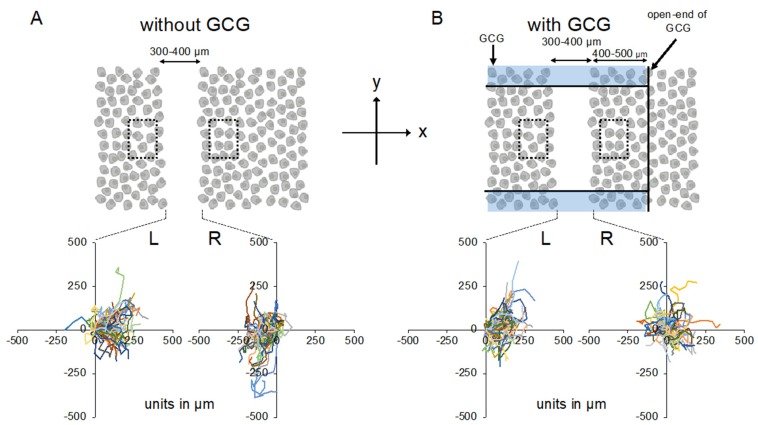
Trajectories of MDA-MB-231 cells measured for 24 h. The oxygen concentration in the microincubator was 21% (room air). Values presented in the graphs are in micrometers. (**A**) Without the GCG placed, cells migrated into the wound space equally from the right (R) and left (L) boundaries of the wound space. (**B**) With the GCG placed, the migration of the cells initially located at the right boundary (R) into the wound space was hindered and they even appeared to migrate in a direction opposite to that toward the wound space as if they were crawling into the crowd of cells. Trajectories of the 50 cells are superimposed. To assess the rate of cell proliferation under GCG, 300 × 500 µm rectangular regions of interest (ROIs) were defined close to but outside the wound space (dashed squares) and the number of cells in the respective ROI was counted and compared at time = 0 and time = 24 h.

We completed five “without GCG” and five “with GCG” experiments, respectively, and representative data associated with these “without GCG” and “with GCG” experiments are shown in Supplementary Video S1 and Video S2, respectively. Figure 3 illustrates the trajectory of individual MDA-MB-231 cells for 24 h.

**Figure 4:**
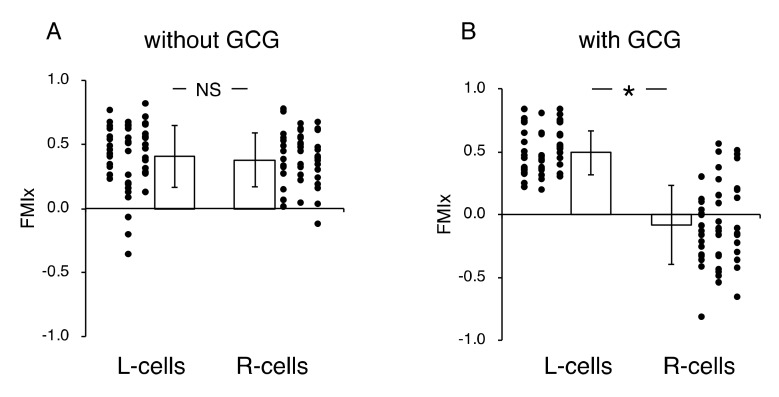
Forward migration index (FMI) parallel to the diffusion path (FMI_x_) without (**A**) and with the GCG in place (**B**). Without the GCG involved, the FMI_x_ values for the L-cells and R-cells were not different (NS, *p* = 0.60). In contrast, with the GCG in place, the FMI_x_ value for the R-cells was significantly smaller compared to that of the L-cells. Data were accumulated from five independent experiments in which 10 cells were sampled in each experiment. Error bars represent the SD. *, *p* < 0.05, as judged by Student’s *t*-test.

Without the GCG, accumulated distances for 24 h in cells migrating into the wound space from the left boundary (L-cells) and from the right boundary (R-cells) were similar (368 ± 94 µm and 358 ± 112 µm, respectively). With the GCG, the accumulated distance was significantly decreased, while values for the L-cells and R-cells were not different from each other (322 ± 118 µm and 295 ± 138 µm, respectively). Without involvement from the GCG, the L-cells and R-cells similarly migrated into the wound space (Figure 3A). Forward migration index (FMI) values were not different between the L-cells and R-cells (Figure 4A). 

In contrast, in the “with GCG” experiments, we demonstrated distinct differences in the direction of cell migration, particularly in the R-cells. With the inclusion of the GCG, the migration of R-cells into the wound space appeared to be considerably hindered (Figure 3B). These cells even migrated in the direction opposite of that toward the wound space, as if they were instead crawling into the crowd of cells. In fact, the FMI value for R-cells was not different from the null (Figure 4B). These data are consistent with that cells under the GCG demonstrate directional migration toward the open-end of the GCG (right side). FMI values perpendicular to the diffusion path (i.e., y-axis) were not different from the null, regardless of GCG involvement. Directional cell movement can be visually confirmed by observing Video S2.

**Figure 5:**
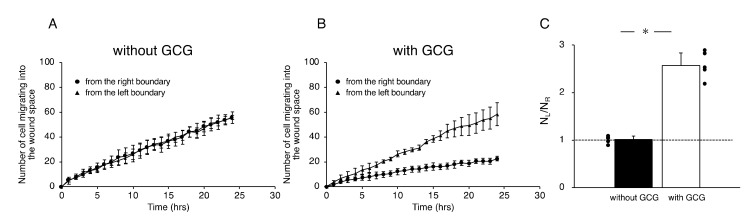
The number of cells that migrated into the wound space in 24 h. (**A**) Without the GCG, the numbers of cells migrating into the wound space from the left boundary and the right boundary were the same. (**B**) With the GCG, the numbers of cells migrating into the wound space from the left boundary and the right boundary were different, with a statistically significant difference demonstrated after five hours. (**C**) The ratio of the numbers of cells migrating into the wound space from the left boundary and the right boundary determined at 24 h (N_L_ and N_R_, respectively). The directional migration of MDA-MB-231 cells was clearly demonstrated with involvement of the GCG. Error bars represent the SD. Data were accumulated from five independent experiments. *, *p* < 0.05, as judged by Student’s *t*-test.

We further checked the directional migration of the cells under GCG by counting the numbers of L-cells and R-cells in the wound space. As shown in Figure 5A, without the GCG in place, the numbers of L-cells and R-cells were the same throughout the experiment. In contrast, with the GCG in place, the numbers of R-cells were smaller than that of the L-cells (Figure 5B), reaching statistical significance after five hours. Thus, the numbers of L-cells and R-cells at 24 h were highly different from one another (Figure 5C). 

Effects of cell proliferations on heterogeneous cell migration into the wound space were also evaluated. Without GCG in place, number of the cell in the dashed square on the L-side and that in the dashed square on the R-side (see Figure 3A) similarly increased in 24 h to 111 ± 11% and 109 ± 8% compared to those at time = 0, respectively, representing proliferation of the cell. With the GCG in place, number of the cell in the dashed square on the L-side counted at 24 h increased to 112 ± 14%, while that in the dashed square on the R-side was 98±16%. The difference was not statistically significant (*p* = 0.055). These results indicate that the difference in the cell proliferation rate in the metabolic gradients had no significant impact on the present wound-healing assays. From these data, we concluded that MDA-MB-231 cells under the GCG demonstrate directional migrations toward the open-end of the GCG.

**Figure 6:**
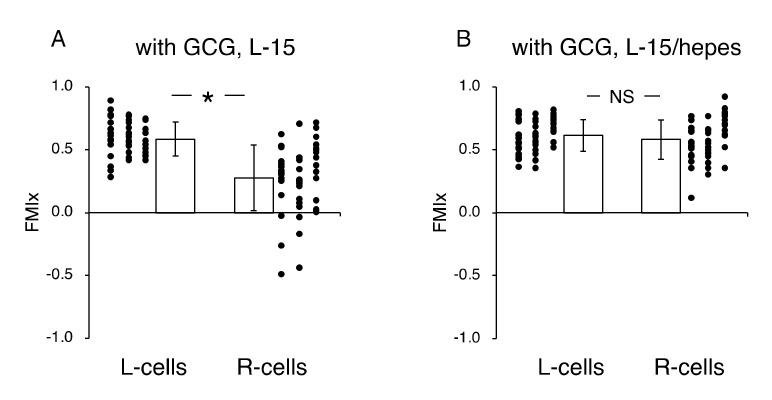
Effects of extracellular pH gradients on FMI_x_ in cells underneath GCG. (**A**) In L-15 medium, the FMI_x_ for the L-cells was significantly higher than that for the R-cells. This result is consistent with that in Figure 4B. (**B**) In L-15/hepes medium in which extracellular pH gradients disappeared, the FMI_x_ values for the L-cells and R-cells were not different (*p* = 0.20), indicating that the directionality in cell migration also disappeared. Data were accumulated from five independent experiments in which 10 cells were sampled in each experiment. Error bars represent the SD. *, *p* < 0.05, as judged by Student’s *t*-test.

Next, we undertook another series of experiments in which the role of extracellular pH gradients in directional movements of MDA-MB-231 cells was examined. We completed five “L-15” and five “L-15/hepes” experiments, respectively. Figure 6 illustrates the analysis of the directionality of cell migration. With the GCG in place, the magnitude of the FMI for the R-cells was significantly smaller than that for the L-cells in L-15 medium (Figure 6A), while FMI values for the L-cells and R-cells were not different from one another in the L-15/hepes medium (Figure 6B). 

**Figure 7:**
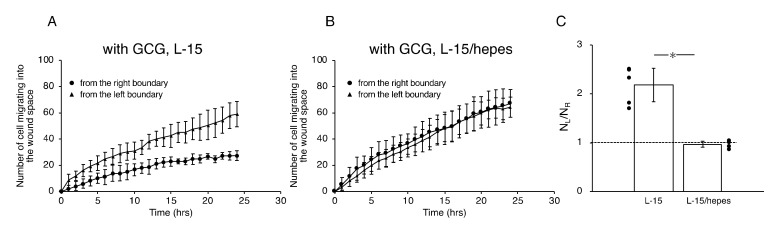
The number of cells that migrated into the wound space in 24 h. (**A**) In L-15 medium, N_L_ and N_R_ were different, consistent with what is observable in Figure 5B. (**B**) Conversely, in L-15/hepes medium, N_L_ and N_R_ were the same. (**C**) The heterogeneity in cell migration ultimately disappeared in L-15/hepes medium. Error bars represent the SD. Data were accumulated from five independent experiments. *, *p* < 0.05, as judged by Student’s *t*-test.

In the L-15 medium, the number of cells migrating from the left boundary into the wound space was significantly higher than that from the right boundary (Figure 7A,C). In comparison, in the L-15/hepes medium, such heterogeneity disappeared (Figure 7B,C). These results indicate that the extracellular pH gradient is the dominant cue for the directional migration of MDA-MB-231 cells under our GCG.

## 3. Discussion

Directional migration of primary cancer cells toward intratumor blood/lymphatic vessels should elevate the probability for intravasation and ultimate hematogenous metastasis. On the analogy of chemotaxis, many presume that the gradients of nutrients and metabolic waste in the local tissue might guide tumor cells to nearby microvessels. However, at the present time, presence of such metabolic cues still remains an open question.

In the present study, we specifically focused on the gradients of H^+^ and oxygen as candidates for the metabolic cue. To monitor migratory behaviors of the cell in gradients of pH and/or oxygen in vitro, we previously proposed a simple microfluidic glassware, GCG, which is capable of producing gradients of energy substrates and metabolites, including H^+^ and oxygen in monolayer cultured cells [11]. Simultaneous changes in H^+^ and oxygen concentrations under the GCG are similar to those found in solid tumors and, therefore, experimentation using the GCG reflects a clinically relevant setting. Unlike the recent microfluidic devices designed for investigating cell migration under oxygen concentration gradients [15,16,17,18], the magnitude of the metabolic gradients under our GCG depends on the metabolic activity of cells per unit volume and cannot be easily manipulated; control of the gradient would require a redesign of the GCG or an accurate adjustment of the cell density (Figure 1C). It is also impossible for our GCG to produce concentration gradients of a specific molecule in the extracellular space. Thus, additional experiments are required to specifically pinpoint the molecule-of-importance among various metabolic substrates/metabolites.

Previous in vitro studies demonstrated the possibility that oxygen concentration gradients may act as a guiding cue for cell migration. Mosadegh et al. [19] used a unique paper-based 3D cell culture system in which oxygen and nutrient gradients were produced along a stack of eight 40-µm-height cell culture layers. They compared metastatic potential in various subtypes of human alveolar adenocarcinoma A549 cells and demonstrated in one A549 subpopulation that oxygen was the primary chemoattractant in their invasion stack; the cells migrated toward the higher oxygen layers. Neither oxygen concentration in each individual layer nor the magnitude of oxygen concentration gradients along the stack was reported. Lewis et al. [20] observed migration of individual sarcoma cells embedded in oxygen-controllable hydrogels in which gradients of oxygen were established. With a 2.5 mm-thick hydrogel (hypoxic gel), the measured oxygen concentrations at the bottom of the hydrogel layer decreased to 0.4%–4% O_2_ from air level corresponding to oxygen gradients ranging from 3.4%–5.4% O_2_/mm. They found that individual sarcoma cells in the hypoxic gel migrate more quickly, across longer distance, in the direction of increasing oxygen concentration compared to those in the normoxic hydrogel with much smaller oxygen gradient. Chang et al. [16] demonstrated using a sophisticated microfluidics platform that under the combination of oxygen and chemokine (SDF-1α) gradients A549 cells migrated toward lower oxygen regions in a 5% O_2_/mm oxygen gradient. Sleeboom et al. [18] demonstrated that both MDA-MB-231 cells and their stem cell enriched population similarly migrate toward low oxygen levels in a 2% O_2_/mm oxygen gradient as determined by cell migration trajectory and the forward migration index. The average local oxygen concentration along the oxygen gradient varied from 1% to 7%, which did not significantly affect the forward migration index of the individual cell. Shih et al. [17] proposed a microfluidic device in which oxygen concentration gradients with a magnitude of 18% O_2_/mm were established along the 900 µm-wide cell channel using oxygen scavenging chemical reactions. HUVECs located at the boundaries of 300 µm-wide cell regions migrated differently in the oxygen gradient; cells initially located at the boundary with higher oxygen concentration in the gradient migrated slowly compared to those with lower oxygen concentration, resulting in a collective cell migration toward lower oxygen.

Results of these in vitro studies appear conflicting in terms of the direction of cell migration in oxygen gradients. However, effects of oxygen gradient on the cell migration should differ according to cell type, culture conditions (2D or 3D, culture media) and spatial profiles of oxygen concentration, including the magnitude and the local oxygen concentration at which cell is exposed. Furthermore, in most microfluidic devices, profile of oxygen concentration gradients may, more or less, change after loading cells in the device due to metabolic activity of the cell. More importantly, metabolic activities of the cell may also change spatial distribution of nutrients and metabolites that may affect the cellular migratory behavior in addition to the effect of the oxygen concentration gradient. 

Initially, we hypothesized that the extracellular gradients of oxygen might be a cue for MDA-MB-231 cells to migrate directionally because steep gradients of oxygen concentration (~10 mmHg/100 µm) have been demonstrated in vivo [5]. Hypoxia-inducible factor 1α (HIF-1α) is an intracellular oxygen sensor that has been reported to affect the intracellular machinery for cell migration [21]. Therefore, it is likely that the HIF-1α pathway plays a role in directing cell migration in the steep gradient of oxygen concentration. However, in the present setting, the direct measurement of the oxygen concentration under the GCG achieved in the present study (Figure 1A) revealed oxygen gradients with relatively small magnitudes such that the oxygen concentration recorded at ~400 µm inside the GCG was much higher (~14%) than the oxygen level at which HIF-1α is responsive (5% or lower [22]). Thus, it is difficult to attribute the directional cell migration demonstrated in the present study to HIF-1α dependent mechanisms. It should be noted here, however, that the present results do not exclude the possibility that oxygen gradients might direct cell migration because the oxygen concentration can drop to pathophysiological levels in hypoxic tumor tissue in vivo [5,23]. Nevertheless, directional cell migration at unphysiologically high oxygen concentrations demonstrated in the present study prompted us to seek another possible cue for directional cell migration. 

A few studies to date have addressed directional cell migration under gradients of extracellular pH in vitro. Paradise et al. [24] demonstrated using the Dunn chamber that both α_V_β_3_ CHO-B2 cells and primary microvascular endothelial cells preferentially migrate toward acid in an extracellular pH gradient. In their research, the Dunn chamber produced a pH gradient of 6.0 to 7.5 over 1 mm. Elsewhere, Jagielska et al. [25] determined that oligodendrocyte precursor cells migrate toward areas of acidic extracellular pH produced by the Zigmond chamber. Here the pH gradient was set to 6.0 to 7.0 over a distance of 1 mm. 

In the current study, we found that, first, a gradient of pH was in fact established in the extracellular medium under the GCG (0.2–0.3 units/mm); second, MDA-MB-231 cells under the GCG preferentially migrated toward the open-end of the GCG (i.e., higher pH/O_2_ regions); and third, such findings of directional cell migration completely disappeared when the extracellular pH gradient was abolished. Albeit, while gradients of various metabolic substances should exist under the GCG, these results strongly indicate that extracellular pH gradient is the predominant cue for the migration of MDA-MB-231 cells under the GCG. 

Among migrating cells, gradients of intracellular and extracellular pH have been demonstrated at the level of the single cell. Martin et al. [26] observed intracellular pH (pH_i_) gradients within single melanoma cells incubated in hepes-buffered Ringer solution at a pH of 7.0 and reported that the mean front-to-rear pH_i_ gradients measured ~0.15 units over a 20-µm distance in migrating human (MV3) and murine (B16V) melanoma cells, where the front (leading edge) was more alkaline. Using a combination of pH-sensitive fluorescent dye and total internal reflection microscopy, Ludwig et al. [27] determined the pericellular pH on the surface of the plasma membrane (pH_em_) in polarized MV3 cells, reporting significant gradients of pH_em_ in single cells where front (at focal adhesions)-to-rear pH_em_ gradients were ~0.2 units (the cell front was more acidic), indicating the existence of nano-domains with distinct pH values on the surface of the plasma membrane. These subcellular heterogeneities in pH_i_ and pH_em_ arise from the heterogeneous distribution of the Na^+^/H^+^ exchanger isoform 1 (NHE1), a major plasma membrane protein that extrude protons from cytosol, where NHE1 accumulates in the leading edge of migrating cells [26,28,29]. Both pH_i_ and pH_em_ gradients may independently affect cell motility through effects on cytoskeletal machinery and cell-matrix interactions, respectively [30]. In fact, a substantial role of NHE1 activities in cell motility has been demonstrated in various cells [28,31,32,33,34].

In the present experiment, we imposed 0.2–0.3 units/mm gradients of pH in the extracellular medium that correspond to a gradient of 0.02 units per single MDA-MB-231 cell. The value is far smaller, if compared at the single-cell level, than the NHE1-driven gradients of pH. This is also true in previous studies in which microfluidic devices produced ~1-unit/mm gradients of pH in the extracellular medium [24,25]. Therefore, it is unclear whether the relationship between cell migration and the NHE1-driven pH_i_ and pH_em_ heterogeneities would be directly applicable to the present and other experiments in which the pH of the extracellular bulk medium was manipulated.

Although there is a possibility that subcellular heterogeneities in pH_i_ and pH_em_ might endow migrating cells with directionality, we are reluctant to conclude that relatively small gradients of extracellular pH at the single-cell level could be a consistent cue for the migration behavior demonstrated in the present study. Instead, we propose a different model of directional cell migration in which stochastic cell movement is modified by macroscopic (spanning a few hundred microns) gradients in the extracellular pH. This model is based on the dependence of cell migration activity on extracellular pH [25,33,34], without assuming significant intracellular pH gradients. Stock et al. [33] demonstrated that the pH of the extracellular bulk solution significantly affects migratory activities in MV3 cells in vitro. At low extracellular pH values, cell-matrix interactions via integrin α_2_β_1_ appear too strong, while they are too weak at high extracellular pH values, both of which hinder migratory activity. Thus, cell migration was most optimally facilitated at an extracellular pH of ~7.0. Based on this bell-shaped extracellular-pH migration-velocity relationship, it is predicted that cells initially located in regions with extracellular pH values of ~7.0 would vigorously move toward either lower or higher pH regions (random directions). As cells happen to migrate into lower or higher pH regions, the migration velocity gradually decreases and, finally, cells would become trapped in the lowest or highest pH regions, respectively. Thus, a degree of heterogeneity in cell distribution across the pH range would be established. It is predictable from this model that the direction of migration (toward the lower or higher pH regions) depends upon the initial extracellular pH, specifically, whether it is lower or higher than the pH at which cell migration is most optimally facilitated. 

In association with hypoxia, acidosis is another metabolic hallmark of solid tumors [9,35]. A type of metabolic reprograming known as the Warburg effect in cancer cells and reduced washout of CO_2_/H^+^ from the tissue are the known major causes of an acidotic microenvironment [36]. In the process of adaptation to acidosis, cancer cells may acquire a malignant phenotype [37,38]. A low pH in the tumor microenvironment in vivo reflects the presence of steep gradients of pH between cells and the blood. If the present results are applicable to in vivo conditions, acidotic tumor cells might preferentially migrate toward more alkaline intratumor vessels. With the concomitant acidotic induction of vascular endothelial growth factor and angiogenesis [39], directional cell migration would elevate the probability of intravasation and, ultimately, metastasis. Thus, targeting acidosis in the tumor microenvironment may have therapeutic rationales from the standpoint of control of hematogenous metastasis.

In summary, the use of novel microfluidic devise GCG produced gradients of pH and oxygen concentration in the extracellular medium in monolayer MDA-MB-231 cells. We clearly demonstrated heterogeneous migration of the cells into the wound space in such a way that cells preferentially migrated in the direction of higher pH/oxygen concentration. Elimination of pH gradients also abolished the directional cell movement under the GCG thus indicating a possibility that extracellular pH gradients are the dominant guiding cue for migration of MDA-MB-231 in the present setting. Because, under GCG, extracellular oxygen concentration remained at unphysiologically high ranges despite the presence of significant gradients, the effect of oxygen concentration gradients on directional migration is still remain to be determined.

## 4. Materials and Methods 

### 4.1. Cells

Human metastatic breast cancer cell line MDA-MB-231 cells were grown in type I collagen coat culture dishes measuring 35 mm in diameter (354456; Corning Inc., Corning, NY, USA) using Leibovitz’s L-15 medium (11415064; Thermo Fisher Scientific, Waltham, MA, USA) supplemented with 10% FCS (HyClone Laboratories, Logan, UT, USA) and antibiotics (A5955; Sigma-Aldrich, St. Louis, MO, USA) in humidified air (37 °C). Cells showed greater than 90% confluence on the fifth day after the passage. Migration experiments were usually conducted on the fourth day after the passage. 

### 4.2. GCG

**Figure 8:**
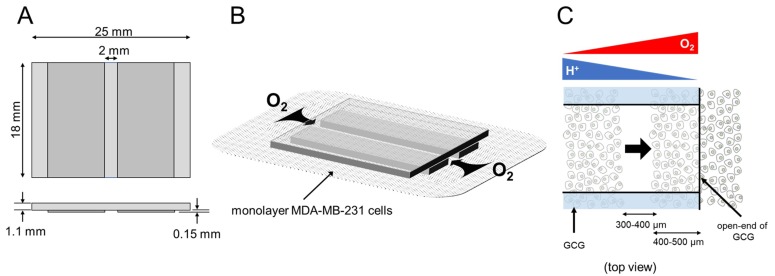
GCG. (**A**) Three thin glass plates were assembled into the GCG. (**B**) The GCG was gently placed onto the monolayer of MDA-MB-231 cells. The diffusional supply of energy substrates such as oxygen to cells in the narrow channel of the GCG is restricted. Thus, cellular oxygen consumption produces gradients of oxygen concentration in the extracellular medium in the narrow channel. Washout of metabolites such as CO_2_/H^+^ to the bulk medium is similarly restricted, and pH gradients are produced in the narrow channel. (**C**) GCG combined with wound-healing assay for cell migration. Gradients of oxygen and H^+^ concentration are also illustrated. In the present study, we demonstrated a vectorial movement of MDA-MB-231 cells toward the open-end of GCG (i.e., higher oxygen concentration or lower H^+^ concentration; indicated by the thick arrow).

To establish metabolic gradients in monolayer cultured cells, we devised a glassware product, GCG [11] (Figure 8). When the GCG is placed on the monolayer cells in a culture dish, oxygen diffusion to the cells in the narrow channel is restricted to only via the two openings, as indicated by the arrows in Figure 8B. Cells in the narrow channel of the GCG consume oxygen. Thus, the balance between the rate of oxygen diffusion into the narrow channel and the rate of cellular respiration is predicted to establish a gradient of oxygen concentration where the oxygen concentration would gradually decrease from the opening of the GCG toward the center of the GCG. Similarly, the restriction of diffusional washout of metabolically produced CO_2_/H^+^ is predicted to establish gradients of extracellular pH, where pH would be higher at the openings (Figure 8C).

### 4.3. Measurements of Oxygen and pH Gradients

We assessed the oxygen concentration gradients in the extracellular medium under GCG by applying an oxygen-sensitive fluorescent foil (SF-RPSu4; PreSens GmbH, Regensburg, Germany) [40]. The oxygen sensor foil consists of layers of transparent polyester support (125-µm thick), oxygen-sensitive layer (6~8-µm thick), and an optical isolation layer made of silicone (20~50-µm thick). The oxygen-sensitive layer emits red fluorescence (wavelength of 655 nm) and green fluorescence (wavelength of 510 nm) upon excitation at 425 nm. The red fluorescence is sensitive to the oxygen concentration and, during calibration using gas with various oxygen concentrations, its magnitude obeyed the Stern–Volmer relationship. Conversely, the green fluorescence is insensitive to oxygen concentration and can be used as a source for the reference fluorescence. We glued a small slip (0.2 × 8 mm) of the foil parallel to the diffusion path in the narrow channel of the GCG. The optical isolation layer was removed. The ratio of the red- and green fluorescence was determined along the oxygen diffusion path and subsequently converted to oxygen concentration according to the Stern–Volmer relationship as determined in separate experiments. We confirmed in every experiment that the fluorescence ratios at ~8 mm inside the GCG and that at ~0.3 mm outside the GCG was close to the ratio values determined at 0% and 21% oxygen gas, respectively.

We also determined the extracellular pH under the GCG using the fluorescent pH indicator dye 3′-O-acetyl-2′,7′-bis(carboxyethyl)-4 or 5-carboxyfluorescein (BCECF; Dojin). BCECF was dissolved in L-15 medium (20 µM). The BCECF fluorescence at 535 nm for the excitation wavelength of 440 nm or 490 nm (F_440_ and F_490_, respectively) was measured and, following background subtraction, the ratio (F_490_/F_440_) was calculated and converted to pH values according to the calibration curve determined separately. Because the thickness of the extracellular medium above the cells was ~0.15 mm, F_490_/F_440_ represents the average pH in this small volume.

All of these measurements were conducted in a culture dish placed on the microscope stage at 37 °C. Fluorescence images were captured with a 14-bit cooled charge-coupled device camera (CoolSNAP MYO; Photometrics, Tucson, AZ, USA) attached to an inverted microscope (IX71; Olympus, Tokyo, Japan). We used a ×2 (PLAPON2X; Olympus, Tokyo, Japan) or ×4 (UPlanFl N; Olympus, Tokyo, Japan) object lens.

### 4.4. Cell Migration Experiments

Among monolayer MDA-MB-231 cells in a culture dish (<90% confluency), a 300- to 400-µm-wide straight wound line was manually produced with a sterilized micropipette tip (Figure 3A). Cells were subsequently washed twice with warm L-15 medium to remove debris. The culture dish was subsequently transferred to the microincubator placed on the stage of the microscope (IX70; Olympus, Tokyo, Japan). After 1.5 h of stabilization, a sterilized GCG was carefully placed on the wound space in such a way that the wound line was perpendicular to the oxygen diffusion path (see Figure 3B and Figure 8C). The distance between one GCG opening and the wound line was 400–500 µm. The oxygen concentration in the microincubator was 20.9% (air) and the temperature was controlled at 37°C using heating glass plates (Kitazato Corp., Shizuoka, Japan) placed on the top and bottom of the microincubator, respectively. The L-15 medium does not contain bicarbonate so the experiment could be conducted in air. Following stabilization for 30 min, phase-contrast images at ×4 magnification were taken every 10 min for 24 h using the sCMOS camera (12-bit, CS-61M; BITRAN Corp., Japan) attached to the microscope and were stored in a computer.

### 4.5. Elimination of Extracellular pH Gradients

Gradients of extracellular pH in GCG were abolished using L-15 medium supplemented with hepes (final concentration 15 mM). The pH was adjusted to ~7.2 using NaOH.

### 4.6. Data Analysis

A total of 144 phase-contrast images were compiled into a stacked TIFF image that was subsequently converted into a movie file. In the initial (time = 0) image, 500 × 300 µm ROIs were defined at the left and right wound boundaries. Ten cells that did not undergo cell division during the 24-h period were arbitrarily selected from the respective ROIs and the relative *x*- and *y*-coordinates were manually determined with 1.5-µm/pixel spatial resolution for each cell every 2 h. Finally, the trajectories of individual cells over 24 h were determined. The accumulated distance of cell migration over 24 h was calculated. Experiments in which the accumulated distance was more than 200 µm within 24 h served for subsequent analyses for directional migration. 

We assessed directionality in cell migration using two different techniques. First, FMI, representing the efficiency of directional cell migration, was calculated from the cell displacement along the diffusion path (*x*-axis) at 24 h divided by the accumulated distance. We compared the average FMI values between the L-cell (cells migrating into the wound space from the left boundary, see Figure 3) and R-cell (cells migrating into the wound space from the right boundary, see Figure 3). Second, we counted the number of L-cells (N_L_) and R-cells (N_R_) in the wound space. If cell migration into the wound space occurs independently of the metabolic gradients in the extracellular space, N_L_ and N_R_ should be the same. On the other hand, if the metabolic gradient in GCG exerts influences on the directionality, then N_L_ and N_R_ should be different. To test this hypothesis, N_L_ and N_R_ were counted every one hour and compared. N_L_ and N_R_ also include the number of proliferating cells in the wound space in addition to that of the migrating cells. To assess the rate of cell proliferation under GCG, 300 × 500 µm rectangular ROIs were defined close to but outside the wound space (dashed squares in Figure 3B) and the number of cells in the respective ROI was counted and compared at time = 0 and time = 24 h. These comparisons were conducted using Student’s *t*-test or Welch’s *t*-test according to the condition defined by Fisher’s *F*-test. Data are presented as mean ±SD. *P*-values less than 0.05 were considered significant.

## Supplementary Materials

Supplementary materials can be found at https://www.mdpi.com/1422-0067/21/7/2565/s1.
